# Does Omega-3 Help After All in Autism Spectrum Disorder? An Update Systematic Review and Meta-Analysis of Randomized Trials

**DOI:** 10.3390/nu18172821

**Published:** 2026-08-28

**Authors:** Inmaculada Sánchez-García, Cristina Sánchez-Quesada

**Affiliations:** 1 Department of Nursing, Faculty of Health Sciences, University of Jaén, 23071 Jaén, Spain; isgarcia@ujaen.es; 2 Preventive Medicine and Public Health Division, Department of Health Sciences, Faculty of Health Sciences, University of Jaén, 23071 Jaén, Spain; 3 University Institute of Research on Olive and Olive Oils (INUO), University of Jaén, 23071 Jaén, Spain; 4 Agrifood Campus of International Excellence (ceiA3), 14071 Córdoba, Spain

**Keywords:** ASD, irritability, omega-3 supplementation, polyunsaturated fatty acids, communication

## Abstract

Background/Objectives: Autism Spectrum Disorder (ASD) affects approximately 60 × 10^4^ incident cases, 283.3 × 10^5^ prevalent cases, and 43.1 × 10^5^ DALYs globally and is frequently accompanied by irritability, hyperactivity, and social withdrawal linked to low-grade neuroinflammation. Omega-3 supplementation is among the most common complementary approaches used by families, but clinical evidence remains inconsistent, and prior meta-analyses have relied on a single outcome instrument. This study aimed to evaluate the efficacy of omega-3 supplementation on ASD-associated behavioral symptoms by harmonizing outcomes across multiple validated instruments. Methods: Twelve randomized controlled trials (number of studies per domain: k = 6–12) were identified through a systematic search of ten databases (CRD420261390092). Outcomes from eight instruments (ABC, CGI-I, BASC-2, SRS/SRS-2, GARS-2, CARS/SDQ, VABS/PLS-4/PDDBI, and CBCL) were mapped onto five behavioral domains: Irritability, Lethargy/Social Withdrawal, Stereotypy, Hyperactivity, and Inappropriate Speech/Communication. Random-effects models (REML, Knapp-Hartung adjustment) pooled Hedges’ *g* for each domain, with fixed-effects, subgroup, and PET-PEESE publication-bias sensitivity analyses. Results: Significant pooled effects favoring omega-3 supplementation were observed for Irritability (*g* = −0.287, 95% CI [−0.562, −0.013], *p = 0.043*) and Inappropriate Speech/Communication (*g* = −0.213, 95% CI [−0.363, −0.063], *p = 0.010*). Lethargy/Social Withdrawal, Stereotypy, and Hyperactivity showed trivial-to-small, non-significant effects. Heterogeneity was negligible (I^2^ ≤ 0.83%) in four domains; Hyperactivity showed moderate heterogeneity (I^2^ = 37.86%). Sensitivity analyses confirmed robustness, and no evidence of publication bias was detected. Conclusions: Expanding synthesis beyond a single outcome instrument revealed statistically significant, though clinically modest, benefits of omega-3 supplementation on irritability and communication symptoms in ASD, effects undetectable in prior smaller, single-instrument analyses. These findings support its consideration as a low-risk adjunctive option, while larger, adequately powered trials remain needed to confirm clinical relevance.

## 1. Introduction

Autism Spectrum Disorder (ASD) is a neurodevelopmental condition defined by deficits in social communication and restricted, repetitive behaviors, with prevalence now estimated at 28 million people [1]. Since the publication of the DSM-5 (Diagnostic and Statistical Manual of Mental Disorders, Fifth Edition), previously distinct diagnoses such as Asperger’s disorder and autistic disorder have been consolidated under a single spectrum diagnosis [2,3]. With recent ADDM Network surveillance indicates a figure attributed to heightened clinical awareness, evolving diagnostic criteria, and potential environmental and nutritional contributors [2,4]. Beyond core symptoms, children with ASD frequently present with irritability, hyperactivity, and social withdrawal, often accompanied by low-grade neuroinflammation characterized by elevated pro-inflammatory cytokines (e.g., IL-1β, IL-6, and TNF-α) increasingly recognized as a key pathogenic mechanism [5,6,7,8] and elevated pro-inflammatory cytokines. Nutritional deficiencies, including reduced serum folate and vitamin B12, are also well documented, often linked to food selectivity [4,9]. Indeed, strong inflammation states are associated with ASD, often linked to immune system dysfunction. Several cell types are enrolled to trigger and sustain these processes (monocytes, macrophages, and microglia cells). Neuro-inflammation and neuro-immune abnormalities have now been established in ASD as key factors in its development and maintenance [10]. Because of this, addressing inflammatory processes could be the aim of the next pharmacological therapy for ASD. Future therapies could also address the changes in neuro-glial responses in the brain and restore neuro-immune alterations.

Omega-3 polyunsaturated fatty acids (DHA and EPA), essential to neuronal membrane function and anti-inflammatory signaling, are consistently reduced in children with ASD relative to typically developing peers. These fatty acids are essential structural components of the central nervous system, critical for neuronal membrane fluidity and anti-inflammatory signaling. Children with ASD consistently show lower circulating DHA and EPA levels compared to typically developing peers, alongside an elevated omega-6 to omega-3 ratio that may contribute to neuroinflammation and impaired brain development [6,7,8]. Given the adverse-effect burden of approved pharmacological treatments, up to 74% of families turn to complementary approaches, with omega-3 supplementation among the most common [7]. However, clinical trial evidence remains inconsistent, and prior meta-analyses have been limited by small sample sizes, mixed omega-3/omega-6 formulations, or reliance on a single outcome instrument [2,3,9].

This study aimed to evaluate the efficacy of omega-3 supplementation on ASD-associated behavioral symptoms—irritability, social withdrawal, stereotypy, hyperactivity, and inappropriate speech—through a systematic review and meta-analysis harmonizing outcomes across multiple validated instruments to maximize the available evidence base beyond prior single-instrument syntheses.

## 2. Materials and Methods

### 2.1. Study Design

This study was conducted as a systematic review and meta-analysis following the Preferred Reporting Items for Systematic Reviews and Meta-Analyses (PRISMA 2020) guidelines [11] (see Supplementary Material Tables S1 and S2). The review protocol was prospectively registered in the International Prospective Register of Systematic Reviews (PROSPERO) under registration number CRD420261390092. The research question was structured according to the PICO framework: Population (diagnosed with ASD according to DMS-IV and DMS-5 criteria), Intervention (omega-3 fatty acids oral supplementation), Comparator (placebo/usual care), and Outcome (assessed through several validated instruments).

### 2.2. Eligibility Criteria

This meta-analysis synthesizes randomized controlled trials evaluating the efficacy of omega-3 supplementation, rather than observational associations between habitual dietary n-3 PUFA intake and ASD status. Studies were eligible for inclusion if they met all of the following criteria: (a) randomized controlled trials; (b) inclusion of at least two independent groups (experimental and control); (c) participants meeting the criteria for ASD (DMS-IV and DMS-5 diagnosed); (d) the intervention consisting of omega-3 fatty acid supplementation; (e) outcomes assessed using a validated scale; and (f) reporting of means and standard deviations (or sufficient data to calculate them) at both pre-test and post-test for each group.

Studies were excluded if they: (a) lacked a control group; (b) used a single-group pre-post design without comparison; (c) did not report sufficient data for effect size calculation; (d) were assessed with a mix of 3-omega and 6-omega fatty acids; or (e) were reviews, case reports, editorials, or conference abstracts without full-text access.

### 2.3. Information Sources and Search Strategy

A comprehensive and systematic electronic search was conducted across the following databases without date restrictions: CENTRAL—Cochrane Central Register of Controlled Trials, Clinicaltrials.gov, Embase, Google Scholar, MEDLINE, PsycINFO, SCIE—Science Citation Index Expanded, SSCI—Social Sciences Citation Index, and Scopus.

The last search was 24 April 2026. To ensure comprehensive coverage, grey literature was also searched through OpenGrey and institutional repositories, and the reference lists of all included studies were manually screened (snowballing).

The search strategy was developed independently by two researchers, combining indexed terms (MeSH and Emtree) with free-text terms. Boolean operators (AND, OR) were used to combine search blocks. The complete search strategy for PubMed and the rest of the databases is presented in Appendix A.1, Appendix A.2 and Appendix A.3. All searches were conducted from 29 March to 27 April of 2026, and results were downloaded and managed using a reference management software (Mendeley Reference Manager v2.149.0., Elsevier, Amsterdam, The Netherlands). Only studies described in English, Spanish, French, or Italian were eligible. An updated search conducted prior to submission yielded no new eligible studies.

### 2.4. Study Selection

**Figure 1:**
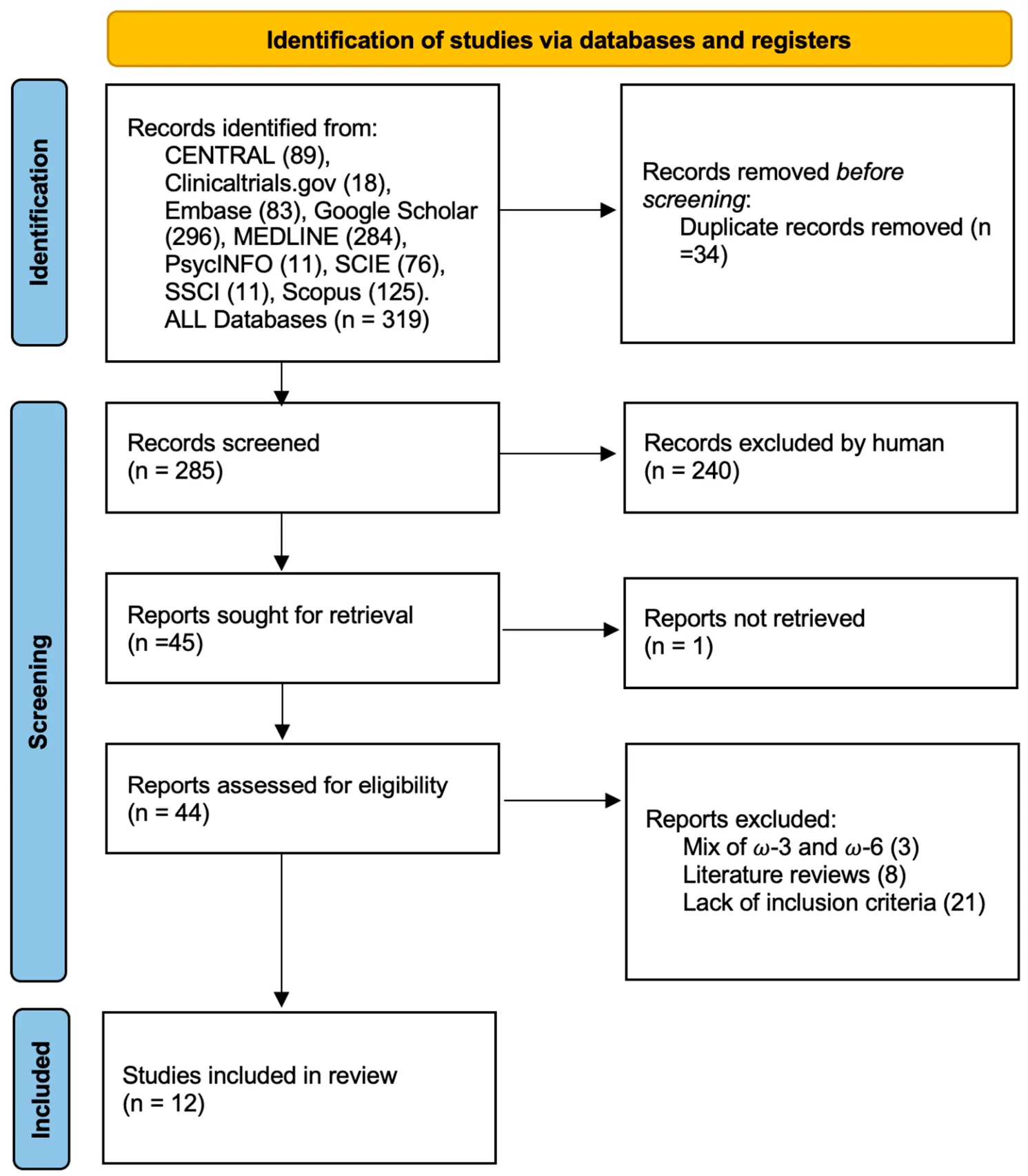
PRISMA 2020 flow diagram.

The selection process was conducted in two independent phases by two reviewers. In Phase 1, titles and abstracts were screened independently and in duplicate by two reviewers, without prior knowledge of each other’s decisions, against the predefined eligibility criteria. In Phase 2, full texts of potentially eligible records were retrieved and independently assessed for inclusion. Disagreements at both phases were resolved by consensus or, when necessary, by consultation with a third reviewer. Inter-rater agreement was quantified using Cohen’s kappa coefficient (κ), with values ≥ 0.70 considered to indicate substantial agreement. The reasons for exclusion at the full-text phase were systematically recorded. The selection process is reported in accordance with the PRISMA 2020 flow diagram (Figure 1).

### 2.5. Data Extraction

A standardized data extraction form was pilot-tested on a random subset of two studies and refined prior to use. Data were independently extracted by two reviewers, with discrepancies resolved by consensus. The following information was extracted from each included study: (a) bibliographic data (first author, year of publication, country); (b) study design and methodological characteristics; (c) participant characteristics (sample size per group, age, sex distribution, diagnostic criteria); (d) description of the intervention and control condition (content, duration, format, setting); (e) outcome measures (instrument name, subscales used, assessment time points); and (f) descriptive statistics—means (M), standard deviations (SD), and sample sizes (n) for both groups at pre-test and post-test. When data were not reported in a usable format, corresponding authors were contacted by electronic mail (up to two attempts with a week’s interval).

Given the heterogeneity of outcome instruments identified during full-text screening, extracted outcomes from non-ABC instruments (CGI-I, BASC-2, SRS/SRS-2, GARS-2, CARS/SDQ, VABS/PLS-4/PDDBI, and CBCL) were mapped a priori onto the five ABC-derived behavioral domains (Irritability, Lethargy/Social Withdrawal, Stereotypy, Hyperactivity, and Inappropriate Speech/Communication) based on conceptual correspondence between subscale definitions, independent of the source instrument. This domain-level harmonization allowed instrument-heterogeneous studies to be pooled within a common behavioral framework rather than restricting synthesis to a single measure. The rationale for pooling outcomes across instruments followed established meta-analytic practice for combining continuous data on a standardized-mean-difference (SMD) scale when studies employ different instruments to measure the same underlying construct [12]. Under this approach, Hedges’ *g* expresses each study’s effect in units of its own within-study variability, permitting comparison across measurement scales without requiring the instruments to be item-equivalent, provided they target a shared behavioral construct. Domain assignment for non-ABC instruments was based on convergence in construct definition and item content with the corresponding ABC domain—for example, ABC Stereotypy and PDDBI Ritualism/Resistance to Change both operationalize restricted, repetitive behavioral patterns, while ABC Irritability and SDQ Conduct Problems both capture aggressive/oppositional behavior. This construct-content correspondence, rather than validated psychometric equivalence (e.g., correlation or factor-analytic convergence between instruments), constituted the basis for mapping decisions, which are detailed in full in Supplementary Table S1. Mapping decisions were made by clinical judgment rather than validated cross-instrument equivalence and are detailed in Supplementary Material (Supplementary Table S1). Global or composite severity indices (e.g., GARS-2 total, PDDBI Autism composite, CGI severity, Behavioral Symptoms Index, and VABS Adaptive Functioning Composite) were treated as global severity measures and excluded from domain-specific pooling, as they do not correspond to a single behavioral construct.

Prior to pooling, the direction of all effect sizes was harmonized so that negative values consistently indicated benefit for the omega-3/intervention group. Instruments in which higher scores denote better functioning (e.g., adaptive-behavior subscales of the VABS and Battelle Developmental Inventory) were sign-inverted to align with the symptom-severity direction used across all other instruments.

For the one study employing a crossover design, only Phase 1 data (pre-crossover) were extracted and analyzed, with the two randomized sequences treated as independent parallel-group arms; this approach avoids potential carryover contamination inherent to crossover designs while preserving a valid between-group comparison at the first assessment point.

The screening of all retrieved records was performed manually and independently by two trained human reviewers at each phase of the selection process. No automated, machine learning-based, or artificial intelligence-assisted screening tools were employed at any stage. This decision was made to ensure full methodological transparency, to minimize the risk of algorithmic misclassification, and to preserve the critical appraisal judgment required for accurate eligibility assessment in this review domain. All decisions were made by the reviewers based solely on the predefined PICO-based eligibility criteria.

### 2.6. Risk of Bias Assessment

The methodological quality of each included study was independently assessed by two reviewers using the Risk of Bias 2 (RoB 2) tool for randomized controlled trials [13]. Each study was rated as having low, some concerns, or high risk of bias across the following domains: randomization process, allocation concealment, deviations from intended intervention, missing outcome data, measurement of the outcome, and selection of the reported result. Disagreements were resolved by discussion and, when necessary, by arbitration by a third reviewer.

### 2.7. Effect Size Calculation

Effect sizes were quantified using Hedges’ *g* (incorporating correction factor *J* [14] across four calculation routes based on data availability: (a) raw mean differences (either change-score or endpoint-only) standardized by the pooled standard deviation. For single-timepoint (endpoint-only) comparisons, SDpooled was computed from endpoint SDs instead of baseline SDs; (b) reconstruction of the standard error from 95% confidence intervals, converted to a *t*-statistic and then to Cohen’s *d*; (c) transformation of odds ratios (OR) to *d* via the Cox method for categorical outcomes; and (d) for crossover trials, a paired within-participant analysis was not performed because neither included trial (Parellada 2017 [15]; Lundbergh 2022 [16]) reported the within-participant correlation coefficient required for this approach, and individual participant data were unavailable; imputing this correlation for standardized mean difference calculation should be undertaken with caution, as it directly affects the magnitude of the effect estimate [12]. Only Phase 1 data (prior to treatment switch) were therefore used, treating the two randomized sequences as independent parallel groups to avoid both an unsubstantiated correlation assumption and potential carryover contamination. Effect sizes were computed via route (a), using endpoint SDs when a true pre-crossover baseline was unavailable. A unified formula was applied across all routes to compute sampling variance (v_i_) and standard error. Finally, scale polarities were harmonized so that a negative *g* consistently indicated a benefit for the intervention (omega-3) group.

### 2.8. Statistical Analysis

#### 2.8.1. Meta-Analytic Model

Analyses were performed using the Classical Meta-Analysis module in JASP (Version 0.96.0.0; JASP Team, 2024). Separate random-effects models were fitted for each of the five ABC-derived behavioral domains (Irritability, Lethargy/Social Withdrawal, Stereotypy, Hyperactivity, and Inappropriate Speech/Communication), pooling effect sizes across the twelve included studies per Section 2.5 domain mapping. Between-study variance (τ^2^) was estimated via Restricted Maximum Likelihood (REML) [17], with the Knapp-Hartung (KNHA) adjustment applied to confidence intervals [18]. Fixed-effects models (inverse variance) were additionally estimated for all five domains as a sensitivity analysis.

#### 2.8.2. Assessment of Heterogeneity

Heterogeneity was quantified using Cochran’s Q (significance threshold *p < 0.10*), the I^2^ index (low < 25%, moderate 25–50%, considerable 50–75%, very high > 75%) [13], and τ^2^ with its 95% CI computed per Viechtbauer (2007) [19]. H^2^ was reported as a complementary variance-ratio measure.

#### 2.8.3. Precision and Prediction

A 95% confidence interval (CI) and a 95% prediction interval (PI) were computed for each pooled estimate [19]. The PI equals the CI when τ^2^ = 0; domains with non-zero τ^2^ yield a wider PI.

#### 2.8.4. Sensitivity Analysis

Sensitivity analyses employed the fixed-effects (inverse variance) model for all five domains. A Baujat plot was generated per domain to identify studies disproportionately contributing to heterogeneity and to the pooled estimate. As an additional sensitivity analysis, the ABC-only subset (k = 4; Amminger 2007 [20], Bent 2011 [21], Bent 2014 [22], Mazahery 2019 [23]) was analyzed separately (Section 3.6) to assess robustness of the expanded multi-instrument pooling. Lundbergh et al. (2022) [16] evaluated adults (mean age of 28 years). We performed a sensitivity analysis excluding this study to examine whether the significant effect observed remains stable or changes in magnitude. According to the standard RoB 2 algorithm, we ran a sensitivity analysis excluding high-risk studies. This will allow us to evaluate whether our core conclusions are reinforced or weakened when restricted to lower-risk trials.

#### 2.8.5. Publication Bias Assessment

Small-study effects were assessed via contour-enhanced funnel plots [24] and the PET-PEESE framework (Precision-Effect Test and Precision-Effect Estimate with Standard Error) [25], regressing observed effect sizes on their standard errors. The test of the standard-error coefficient (PET) served as the primary indicator of funnel plot asymmetry, analogous to Egger’s regression test for each domain. Given limited statistical power with fewer than ten studies in some domains [26], results were interpreted with caution. A *p*-value < 0.10 was used as the asymmetry significance threshold.

#### 2.8.6. Statistical Significance and Effect Size Interpretation

Significance for pooled effects was set at α = 0.05. Effect size magnitude followed Cohen’s (1988) benchmarks [27]: *g* < 0.20 is trivial, 0.20–0.49 is small, 0.50–0.79 is medium, and ≥0.80 is large. All estimates are reported with 95% CI and 95% PI.

#### 2.8.7. Subgroup Analysis

Exploratory mixed-effects meta-regressions examined three moderators—intervention dose (<700 vs. ≥700 mg DHA/day) [28], participant age, distinguishing preschool-aged (<6 vs. ≥6 years) consistent with developmental classifications in the DSM-5, and duration (≤12 vs. >12 weeks)—for each of the five domains, using REML with the Knapp-Hartung adjustment and Q_M for between-subgroup comparisons (α = 0.05).

Generative artificial intelligence (GenAI) has been used in this paper in order to proofread and improve language and readability. Data is publicly available by request to the corresponding author’s email.

## 3. Results

### 3.1. StudiesSelection

The systematic search across all databases retrieved a total of 319 records. After removal of 34 duplicated studies, 285 records were screened at the title and abstract level, of which 274 were excluded based on the predefined eligibility criteria. A total of 44 full-text articles were assessed for eligibility, and 32 were subsequently excluded with documented reasons (no placebo in control groups, no use of omega-3 and omega-6 mix as intervention treatments, or not being ECA articles). Ultimately, twelve studies met all inclusion criteria and were incorporated into the systematic review and meta-analysis. The study selection process is detailed in the PRISMA 2020 flow diagram (Figure 1). Inter-rater agreement for the screening process was substantial (κ = 0.82).

### 3.2. Study Characteristics

Twelve studies were included, published between 2007 and 2022, and conducted across Austria, the USA, New Zealand, Canada, Spain, Iran, and Denmark. All employed a randomized, placebo-controlled design, with the exception of Parellada 2017 [15] and Lundbergh 2022 [16], which used a crossover design (Phase 1 data only analyzed, as detailed in Section 2.7). Sample sizes ranged from 13 to 68 participants. The mean age ranged from 3.23 to 28 years. Omega-3 dosage ranged from 200 mg to 5.2 g/day (EPA + DHA), and intervention duration ranged from 4 to 52 weeks. Placebo conditions varied across studies (coconut, safflower, corn/soybean, olive, sunflower oil, liquid paraffin, or dietary advice). Outcome instruments comprised ABC, CGI-I, BASC-2, SRS/SRS-2, GARS-2, CARS/SDQ, VABS/PLS-4/PDDBI, and CBCL. Descriptive characteristics of each included study are presented in Table 1.

### 3.3. Results of Risk of Bias Assessment

Using RoB 2, six studies were judged low risk overall across all domains (Bent 2011 [21], Bent 2014 [22], Parellada 2017 [15], Mazahery 2020 [23], Doaei 2021 [31], Lundbergh 2022 [16]). Five studies raised some concerns: Amminger 2007 [20] (randomization process); Voigt 2014 [30] and De la Torre-Aguilar 2022 [28] (selection of the reported result); Mankad 2015 [5] (missing outcome data and selection of the reported result); and Mazahery 2019 [23] (missing outcome data, reflecting 34% attrition with higher baseline severity among non-completers). Johnson 2010 [29] was judged overall high risk, driven by deviations from intended interventions and outcome measurement.

Across the twelve included studies, selection of the reported result was the most frequently affected domain, raising concerns in three studies (Voigt 2014 [30], Mankad 2015 [5], De la Torre-Aguilar 2022 [28]), followed by missing outcome data, affecting two studies (Mankad 2015 [5], Mazahery 2019 [23]). The randomization process was well controlled overall, with only one study (Amminger 2007 [20]) raising concerns, largely attributable to incomplete reporting of allocation procedures rather than confirmed deficiencies. Deviations from intended interventions and measurement of the outcome were the domains driving the sole high-risk judgment (Johnson 2010 [29]), reflecting protocol deviations and outcome-assessment concerns specific to that trial.

**Figure 2:**
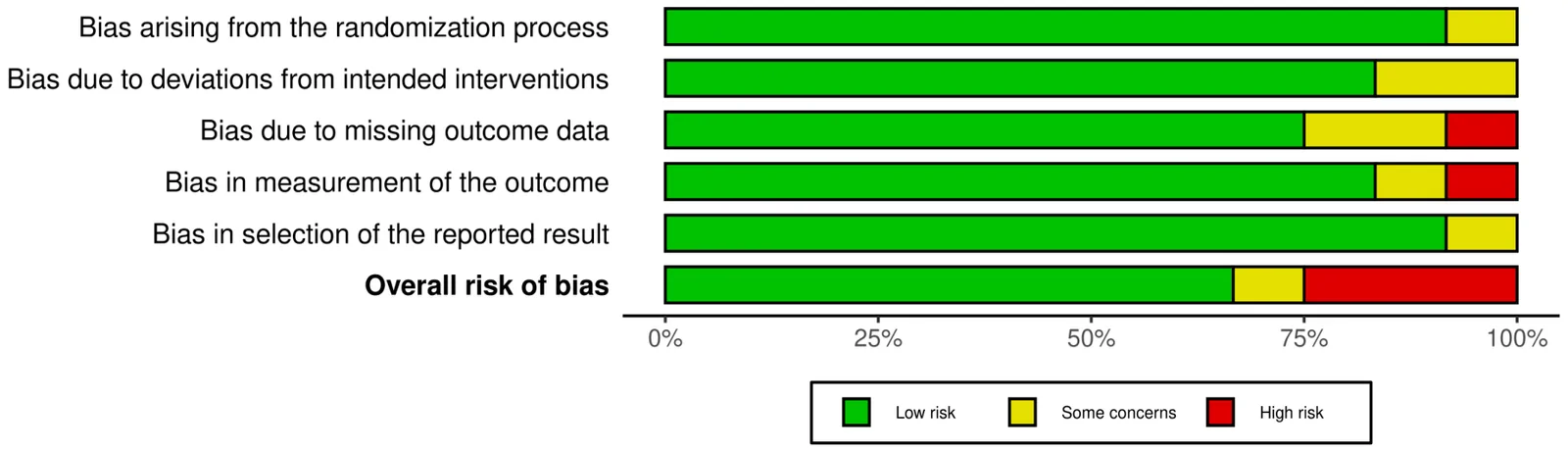
Summary plot of RoB 2 tool.

Overall, half of the included studies (6/12) were free of risk-of-bias concerns across all domains, and the remaining six studies were affected predominantly by reporting- and attrition-related domains rather than by fundamental deficiencies in randomization or intervention delivery—supporting reasonable confidence in the internal validity of the pooled evidence base while warranting the domain-specific sensitivity considerations already discussed in Section 3.6 and Section 4.2 for the studies flagged as high- or some-concerns risk. A summary plot is shown in Figure 2, and a traffic light plot is included in Supplementary Material (Supplementary Figure S1).

### 3.4. Primary Analysis: Expanded Multi-Instrument Metanalysis

Twelve studies employing eight validated instruments (ABC, CGI-I, BASC-2, SRS/SRS-2, GARS-2, CARS/SDQ, VABS/PLS-4/PDDBI, and CBCL) were pooled by behavioral domain, following the mapping strategy described in Section 2.2. Random-effects models (REML, Knapp-Hartung adjustment) were fitted separately for each of the five ABC-derived domains.

#### 3.4.1. Irritability

**Figure 3:**
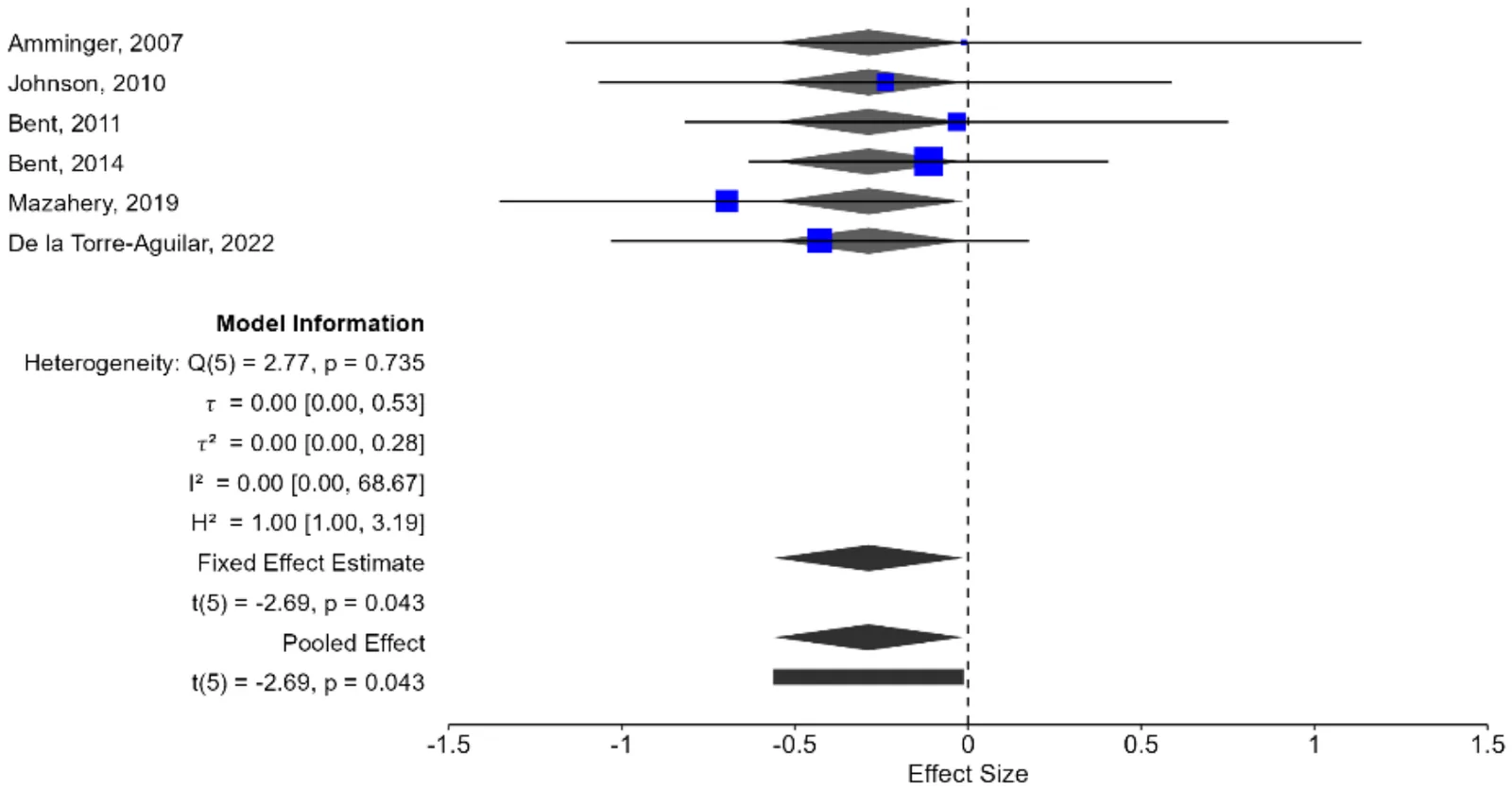
Irritability Forest Plot. Blue square as estimate of the effect size [20,21,22,23,28,29].

Six studies contributed data (k = 6). The pooled effect was Hedges’ *g* = −0.287 (SE = 0.107; 95% CI: −0.562 to −0.013; 95% PI: identical to CI; t(5) = −2.69, *p* = *0.043*), indicating a statistically significant small-to-medium effect favoring omega-3 supplementation. Heterogeneity was negligible (I^2^ = 0.00%; Q(5) = 2.77, *p* = 0.735; τ^2^ = 0.000) (Figure 3).

#### 3.4.2. Lethargy/Social Withdrawal

**Figure 4:**
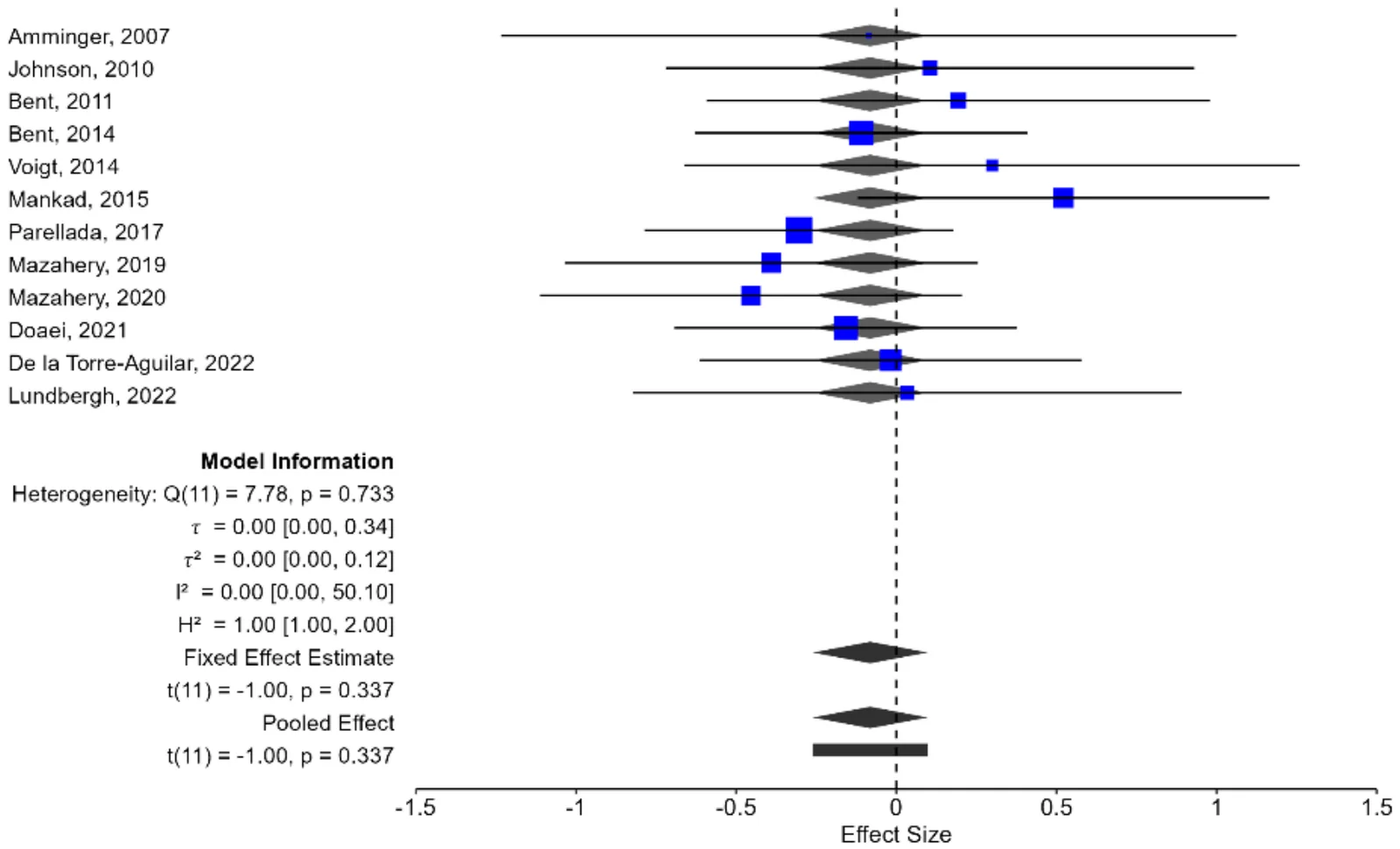
Lethargy/Social Withdrawal Forest Plot. Blue square as estimate of the effect size [5,15,16,20,21,22,23,28,29,30,31].

Twelve studies contributed data (k = 12), the largest domain. The pooled effect was g = −0.082 (SE = 0.081; 95% CI: −0.261 to 0.098; t(11) = −1.00, *p* = 0.337), a trivial non-significant effect. Heterogeneity was negligible (I^2^ = 0.00%; Q(11) = 7.78, *p* = 0.733) (Figure 4).

#### 3.4.3. Hyperactivity

**Figure 5:**
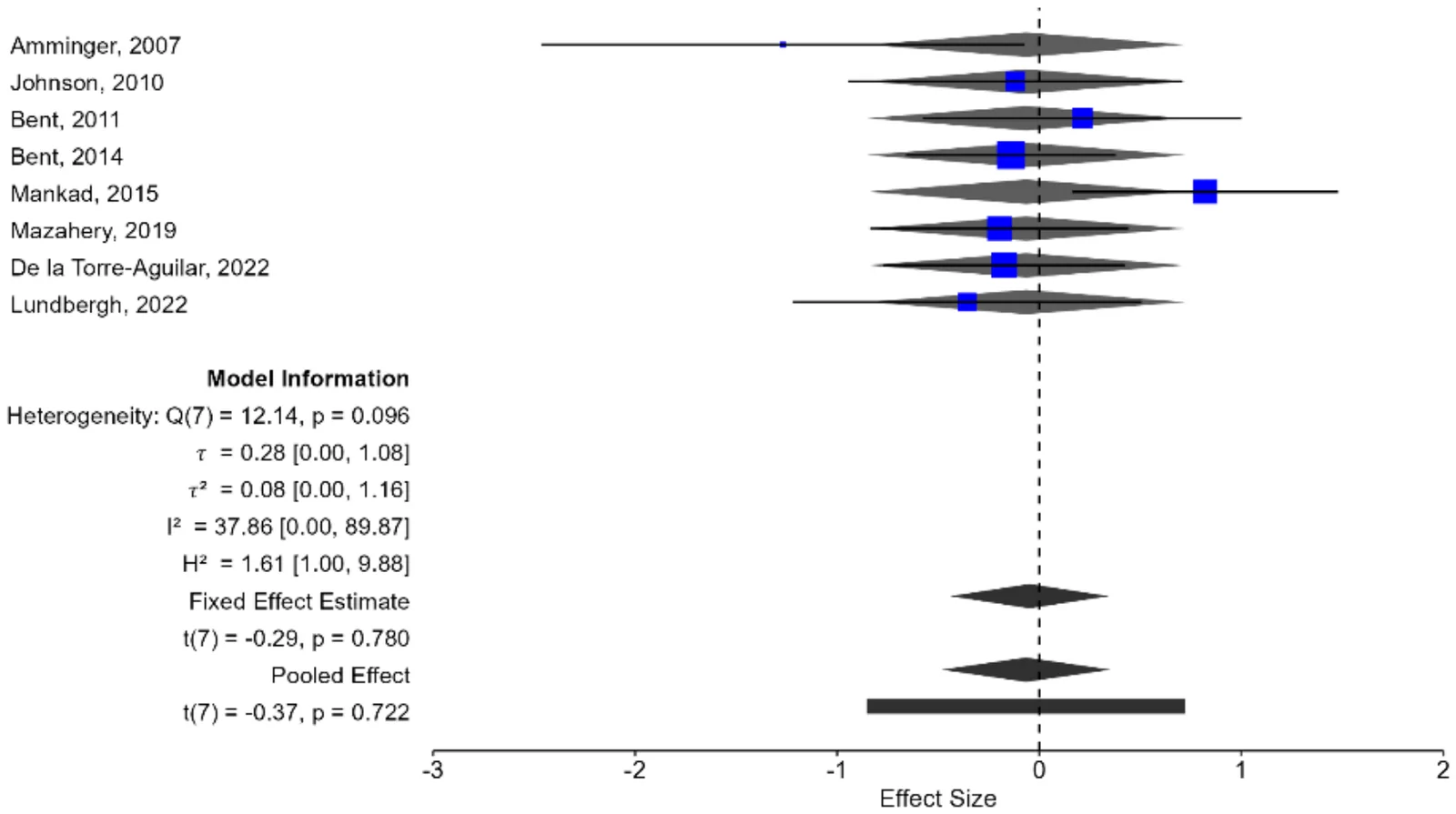
Hyperactivity Forest Plot. Blue square as estimate of the effect size [5,16,20,21,22,23,28,29].

Eight studies contributed data (k = 8). The pooled effect was *g* = −0.066 (SE = 0.177; 95% CI: −0.485 to 0.354; 95% PI: −0.853 to 0.722; t(7) = −0.37, *p* = 0.722), a trivial non-significant effect. Unlike the other four domains, moderate heterogeneity was observed (I^2^ = 37.86%; τ^2^ = 0.080; Q(7) = 12.14, *p* = 0.096), reflected in a considerably wider prediction interval than confidence interval (Figure 5).

#### 3.4.4. Stereotypy

**Figure 6:**
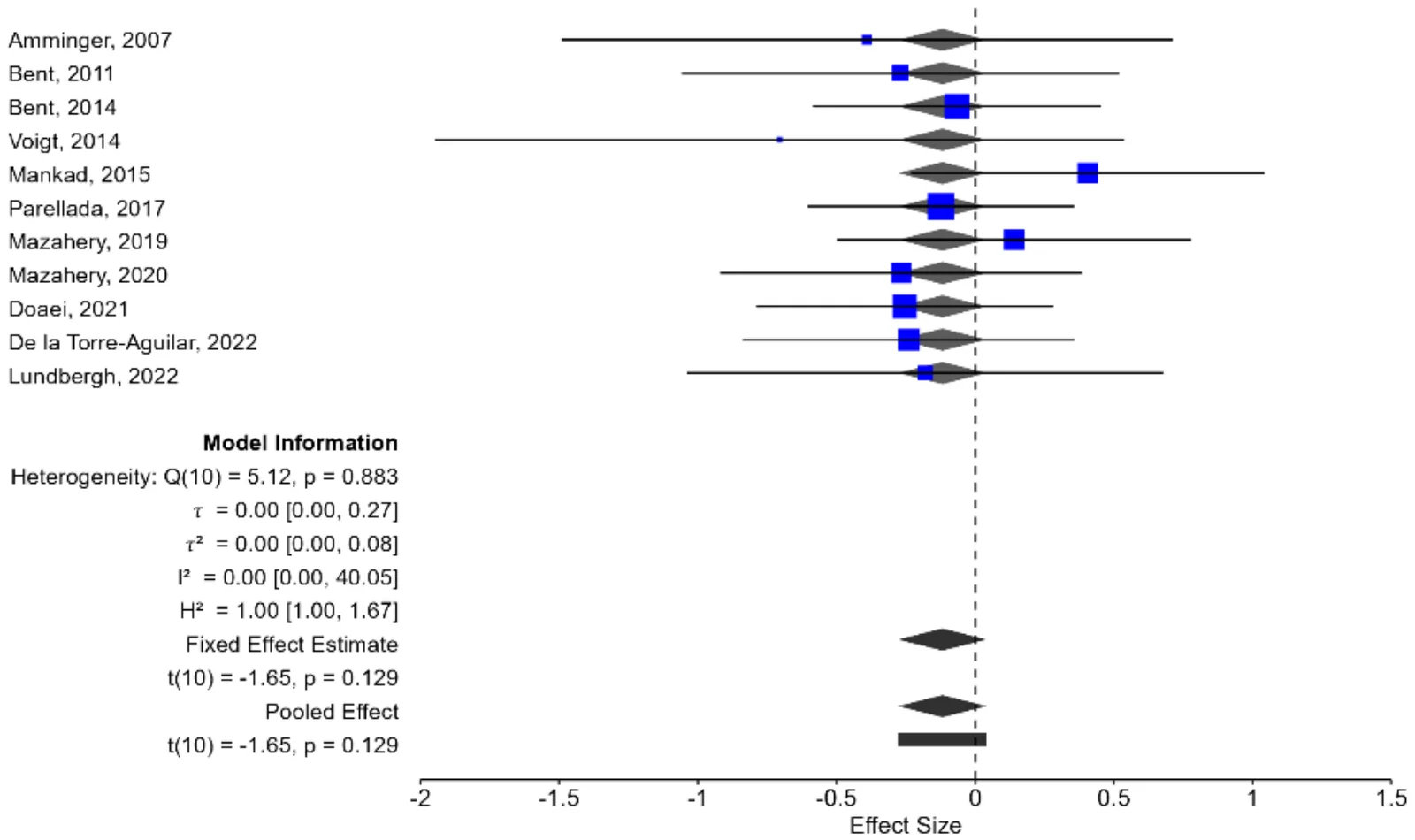
Stereotypy Forest Plot. Blue square as estimate of the effect size [5,15,16,20,21,22,23,28,30,31].

Eleven studies contributed data (k = 11). The pooled effect was *g* = −0.118 (SE = 0.072; 95% CI: −0.278 to 0.041; t(10) = −1.65, *p* = 0.129), a small non-significant effect. Heterogeneity was negligible (I^2^ = 0.00%; Q(10) = 5.12, *p* = 0.883) (Figure 6).

#### 3.4.5. Inappropriate Speech

**Figure 7:**
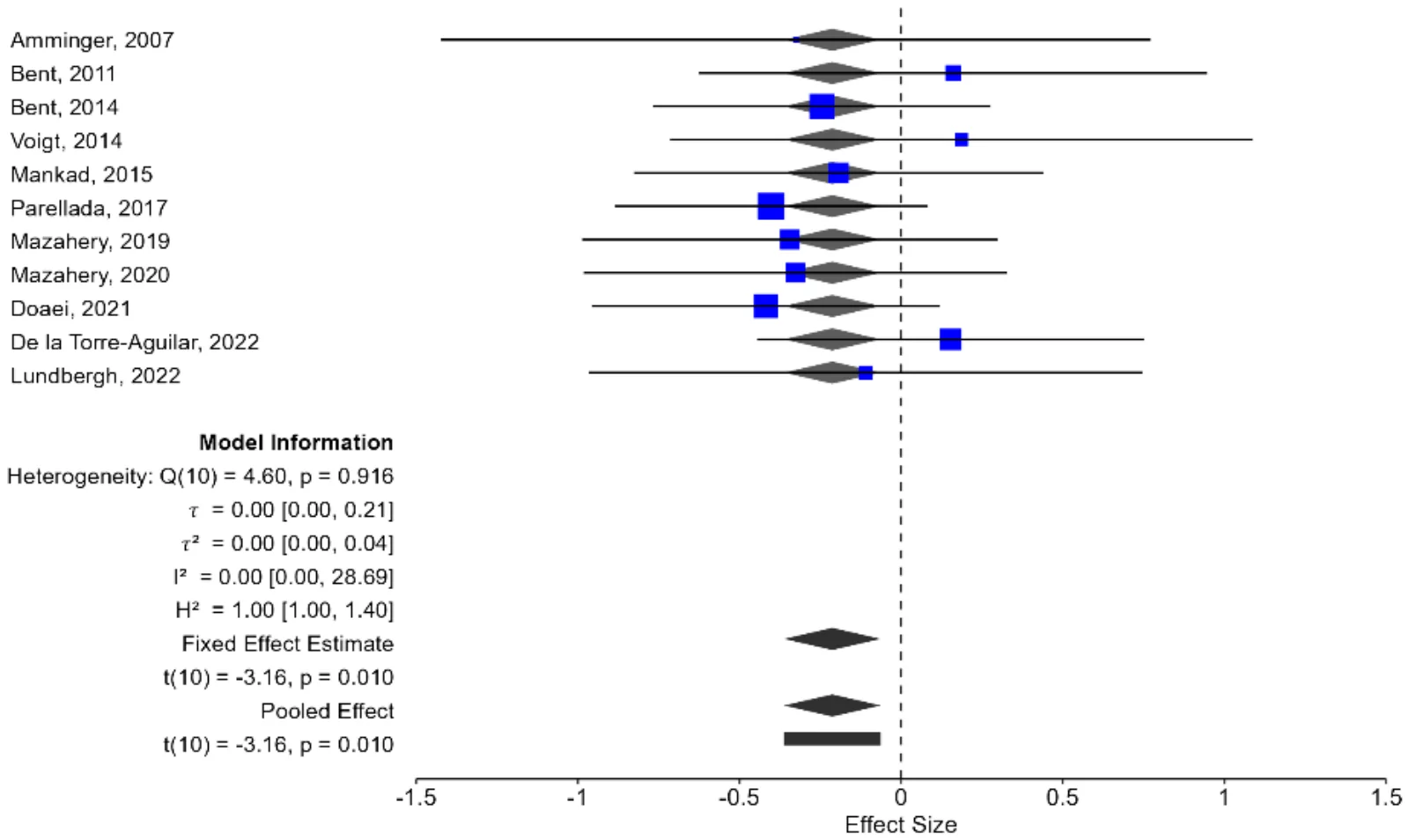
Inappropriate Speech Forest Plot. Blue square as estimate of the effect size [5,15,16,20,21,22,23,28,30,31].

Eleven studies contributed data (k = 11). The pooled effect was *g* = −0.213 (SE = 0.067; 95% CI: −0.363 to −0.063; t(10) = −3.16, *p* = 0.010), a small, statistically significant effect favoring omega-3 supplementation. Heterogeneity was negligible (I^2^ = 0.00%; Q(10) = 4.60, *p* = 0.916) (Figure 7).

### 3.5. Subgroup Metanalysis

Exploratory subgroup meta-regressions examined intervention dose, age, and duration as moderators for each domain (Table 2). For irritability, all three moderators reached statistical significance: dose (Q_M = 4.84, *p = 0.028*), age (Q_M = 4.27, *p = 0.039*), and duration (Q_M = 4.27, *p = 0.039*). For hyperactivity, the dose approached significance (Q_M = 3.03, *p* = 0.082), while age and duration did not (Q_M = 1.99, *p* = 0.158). For Inappropriate Speech, duration was significant (Q_M = 4.39, *p = 0.036*), whereas dose and age were not (*p* = 0.576 and *p* = 0.214, respectively). No moderator reached significance for Lethargy/Social Withdrawal or Stereotypy (all *p* > 0.18). Given the exploratory nature of these analyses and the modest number of studies per subgroup, findings should be interpreted as hypothesis-generating rather than confirmatory.

### 3.6. Sensitivity Analysis Results

**Fixed-effects model.** Fixed-effects models (inverse variance) were additionally fitted for all five domains on the expanded (k = 12) dataset (Table 3). Point estimates were almost identical to the random-effects primary analysis across all domains (Table 3), consistent with the negligible heterogeneity observed in four of the five domains (I^2^ = 0% for Irritability, Lethargy/Social Withdrawal, Stereotypy, and Inappropriate Speech). The random-effects model was retained as primary given its more conservative and generalizable inference and because it remains appropriate regardless of the level of heterogeneity detected [32].

**ABC-only subset (k = 4).** To assess whether the expanded multi-instrument pooling altered conclusions relative to the original ABC-restricted evidence base, the four original ABC studies (Amminger 2007 [20], Bent 2011 [21], Bent 2014 [22], and Mazahery 2019 [23]) were analyzed separately under the same random-effects specification (Supplementary Table S2 and Supplementary Figures S2–S6). All ABC-only estimates were non-significant, with confidence intervals crossing zero across all five domains. Notably, Irritability and Inappropriate Speech—the two domains reaching significance in the expanded 12-study analysis—showed materially similar point estimates in the ABC-only subset (*g* = −0.249 and −0.204, respectively) but failed to reach significance due to substantially wider confidence intervals, reflecting the limited precision afforded by k = 4. This indicates that the expanded analysis did not alter the direction or approximate magnitude of effect but rather increased precision sufficiently to detect effects consistent with, though previously undetectable in, the original evidence base.

Baujat plots identified no single study exerting disproportionate influence on the pooled estimate in most domains. For hyperactivity, the only domain with non-negligible heterogeneity (I^2^ = 37.86%, τ^2^ = 0.080), individual-study contributions were more variable, consistent with the wider prediction interval observed for this outcome (Section 3.4.3); nonetheless, fixed- and random-effects estimates remained closely aligned (*g* = −0.05 vs. −0.066).

A directionally divergent result was noted for De la Torre-Aguilar (2022) [28] within the Inappropriate Speech domain (+0.153), the only study in this domain favoring the placebo group. This is consistent with the original report, which described a significant improvement in Battelle receptive, expressive, and communication scores occurring in the placebo group rather than the treatment group. This isolated directional discrepancy likely reflects a genuine study-level finding rather than a data extraction artifact and is noted here as a potential source of directional heterogeneity within the Inappropriate Speech domain, despite the low overall I^2^ for this outcome.

**Exclusion of the adult population**. Lundbergh et al. (2022) [16] evaluated adults (mean age of 28 years) (Supplementary Table S4). Inappropriate Speech/Communication: the pooled intervention effect remained statistically significant (t = −3.02, *p* = *0.014*), confirming that the beneficial effect on communication symptoms is robustly present within the pediatric population. Irritability: The pooled effect remained unchanged and significant (t = −2.69, *p* = *0.043*), as Lundbergh et al. (2022) [16] did not contribute data to this domain. No significant changes in significance or direction were observed for the remaining domains: Lethargy/Social Withdrawal (t = −1.01, *p* = 0.338), Hyperactivity (t = −0.19, *p* = 0.859), and Stereotypy (t = −1.49, *p* = 0.171).

**Correction of Risk of Bias Classification**. Following a strict algorithmic interpretation of the Risk of Bias 2 (RoB 2) tool, three trials were classified as having an overall high risk of bias (Mankad et al., 2015 [5]; Johnson et al., 2010 [29]; Amminger et al., 2007 [20]). We ran a sensitivity analysis excluding these three studies to assess the stability of our findings when restricted exclusively to trials with lower risk of bias. Irritability: The statistical significance observed in the primary analysis was lost (t = −2.17, *p* = 0.118) when high-risk trials were excluded, suggesting that the pooled effect on irritability is sensitive to methodological quality and should be interpreted with caution. Inappropriate Speech/Communication: Conversely, the intervention effect remained statistically significant (t = −2.64, *p* = *0.030*), confirming the high robustness of omega-3 benefits on communication symptoms. Stereotypy: Interestingly, excluding the high-risk trials unmasked a statistically significant benefit favoring the omega-3 group (t = −3.01, *p* = *0.017*), which was non-significant in our primary analysis (*p* = 0.129). Lethargy/Social Withdrawal approached but did not cross the significance threshold (t = −2.17, *p* = 0.062), while Hyperactivity remained non-significant (t = −1.79, *p* = 0.147).

### 3.7. Publication Bias

Small-study effects were assessed via the PET-PEESE framework, using the test of the standard-error coefficient as an Egger-type indicator of funnel plot asymmetry (Supplementary Table S3). No domain showed evidence of publication bias (all *p* > 0.10): Irritability (t = 0.419, df = 4, *p* = 0.696); Lethargy/Social Withdrawal (t = 1.329, df = 10, *p* = 0.213); Hyperactivity (t = −1.272, df = 4, *p* = 0.272); Stereotypy (t = −1.159, df = 9, *p* = 0.276); and Inappropriate Speech (t = 1.527, df = 9, *p* = 0.161). Given the modest number of studies per domain (k = 6–12), these results should be interpreted with caution, as statistical tests for publication bias have limited power under these conditions [26]. PET and PEESE bias-corrected estimates remained directionally consistent with the primary random-effects estimates for Irritability, Lethargy/Social Withdrawal, and Inappropriate Speech, with the corrected estimate for Inappropriate Speech remaining statistically significant under PEESE correction (*g* = −0.379, *p* = *0.026*). For Hyperactivity and Stereotypy, PET and PEESE corrected estimates reversed sign relative to the primary random-effects estimates; given the small k and, for Hyperactivity, the non-negligible between-study heterogeneity (I^2^ = 37.86%) in these domains, this sign reversal likely reflects estimator instability under bias correction rather than genuine evidence of an opposite true effect and should be interpreted with caution. Overall, these analyses provide no indication that the pooled effects were substantially inflated by small-study or publication bias.

## 4. Discussion

Currently, there is no curative intervention for ASD; existing treatments primarily target associated behavioral symptoms. Only risperidone and aripiprazole hold FDA approval specifically for irritability in ASD [5,9], and both carry substantial adverse-effect burdens (weight gain, sedation, metabolic dysregulation), driving up to 74% of families toward complementary approaches such as omega-3 supplementation [7].

Basic research firmly establishes a pathological basis for omega-3 dysregulation in ASD, characterized by abnormal fatty acid metabolism, accelerated lipid peroxidation, and compromised cell membrane fluidity. This metabolic bottleneck directly impacts synaptic transmission, neuroplasticity, and notably, while dietary patterns in both parents and children strongly influence brain development, evidence from neurodevelopmental research [33] demonstrates that nutrient deficiencies stem from a complex interplay beyond simple habitual eating habits. Maternal seafood consumption during pregnancy and childhood dietary intake illustrate how inadequate intake (“eat less”) can limit essential fatty acids like DHA and EPA, increasing vulnerability to neurodevelopmental disorders. However, clinical data reveals that nutrient deficiency is equally driven by increased physiological demand or altered metabolic utilization (“use more”). Youth with neurodevelopmental conditions frequently exhibit significantly lower red blood cell levels of DHA, EPA, and arachidonic acid (AA) compared to healthy controls, even in cases where dietary intake is similar, pointing toward impaired enzymatic conversion or heightened oxidative consumption. Consequently, evaluating both regional dietary patterns—which shape baseline nutrient intake—and physiological metabolic utilization is crucial for understanding nutrient deficiencies and tailoring targeted, biomarker-guided nutritional strategies. The weak therapeutic effects and positive trends observed across several clinical trials are biologically rational when considering the vast phenotypic and genotypic heterogeneity of the autism spectrum. Rather than indicating a lack of therapeutic value, the modest clinical effect sizes likely reflect a translational gap, wherein a subset of children with profound baseline PUFA deficiencies or specific metabolic profiles benefit significantly, but their response is diluted in broad, unselected cohorts. Therefore, future clinical research should pivot away from broad-spectrum trials and move toward a precision-medicine framework. Clinical evidence must be harmonized with basic science by utilizing baseline fatty acid profiling and biomarkers of inflammation to identify the specific subgroups of ASD children for whom targeting fatty acid metabolism yields the greatest therapeutic efficacy.

This systematic review and meta-analysis synthesized evidence from twelve randomized controlled trials evaluating omega-3 supplementation across five ASD-relevant behavioral domains from eight validated instruments (ABC, CGI-I, BASC-2, SRS/SRS-2, GARS-2, CARS/SDQ, VABS/PLS-4/PDDBI, and CBCL) onto a common domain framework. This expansion increased the evidence base three- to six-fold across three domains (Lethargy/Social Withdrawal, Stereotypy, and Inappropriate Speech: k = 11–12) and more modestly for Irritability (k = 6) and Hyperactivity (k = 8).

Unlike the ABC-only analysis, which found no statistically significant pooled effects, the expanded analysis identified two domains reaching conventional significance: Irritability (*g* = −0.287, 95% CI [−0.562, −0.013], *p* = *0.043*) and Inappropriate Speech/Communication (*g* = −0.213, 95% CI [−0.363, −0.063], *p* = *0.010*), both favoring omega-3 supplementation. Critically, the point estimates for these two domains in the ABC-only subset (*g* = −0.249 and −0.204, respectively) were already directionally and quantitatively similar to the expanded estimates; what changed was precision, not direction. This pattern indicates that the small, non-significant ABC-only findings reflected underpowering rather than the absence of a true effect—the expanded synthesis did not manufacture a new effect but rather resolved one that the four-study evidence base lacked the precision to detect. The remaining three domains (Lethargy/Social Withdrawal, Stereotypy, and Hyperactivity) remained non-significant, with effect magnitudes in the trivial-to-small range (*g* = −0.066 to −0.118).

Heterogeneity was negligible (I^2^ = 0%) in four of the five domains, reinforcing the reliability of the pooled estimates despite substantial clinical diversity in dosage (200 mg–5.2 g/day), duration (4–52 weeks), age range, and outcome instrument. Hyperactivity was the exception (I^2^ = 37.86%, τ^2^ = 0.080), with a correspondingly wide prediction interval and unstable PET-PEESE-corrected estimates, suggesting this domain is the least consistently characterized across the current evidence base and warrants cautious interpretation.

Exploratory subgroup analyses provided a plausible explanation for the pattern of significance: for Irritability, all three moderators (dose, age, and duration) reached significance, suggesting the pooled effect may be driven disproportionately by specific intervention profiles (e.g., higher-dose or longer-duration protocols) rather than reflecting a uniform effect across all trial designs. For Inappropriate Speech, only duration was significant, pointing to a possible dose-independent but time-dependent mechanism. No moderator was significant for Lethargy/Social Withdrawal or Stereotypy. Given the small number of studies per subgroup, these findings remain hypothesis-generating and should not be used to guide clinical dosing decisions.

Sensitivity analyses supported the robustness of the primary findings. Fixed-effects estimates were virtually identical to random-effects estimates across all domains (Δg < 0.01), consistent with the low heterogeneity observed. PET-PEESE publication-bias correction showed no evidence of small-study effects for any domain, and the corrected estimate for Inappropriate Speech remained significant under PEESE (*g* = −0.379, *p* = 0.026), further supporting that this finding is unlikely to be an artifact of selective reporting. The corrected estimates for Hyperactivity and Stereotypy reversed sign relative to the primary estimates, likely reflecting estimator instability given their smaller k and, for Hyperactivity, its elevated heterogeneity, rather than genuine evidence against the observed direction of effect.

One study-level observation merits explicit note: De la Torre-Aguilar (2022) [28] was the sole contributor to the Inappropriate Speech domain favoring the placebo group, consistent with that trial’s own report of significant improvements in Battelle receptive, expressive, and communication scores occurring in the placebo rather than the treatment arm. This isolated divergence did not undermine the pooled significant effect but represents a plausible source of true between-study variability in communication-related outcomes, meriting attention in future primary research.

Clinically, the magnitude of the significant pooled effects (*g* = −0.21 to −0.29) remains modest, corresponding to trivial-to-small effects under conventional benchmarks [27], and considerably smaller than those reported for FDA-approved pharmacological agents for irritability (risperidone *g* ≈ 0.90–1.20; aripiprazole *g* ≈ 0.80–1.00) [34,35,36]. Given the substantially more favorable safety profile of omega-3 supplementation relative to antipsychotics as established in the broader pharmacological and nutritional literature [34,35,36], even a modest but statistically robust effect may hold practical relevance as a lower-risk adjunctive option, particularly for families seeking alternatives to pharmacological treatment. It should be noted that the present meta-analysis did not systematically extract or synthesize adverse-event data from the included trials; this safety comparison is therefore drawn from external literature rather than from a within-review safety analysis and should be interpreted accordingly.

These findings extend and partially revise the conclusions of prior syntheses. James et al. (2011) [3], based on two RCTs, found no evidence supporting omega-3 for ASD symptoms; Cheng et al. (2017) [2], using mixed omega-3/omega-6 formulations without small-sample correction, reported improvements in hyperactivity, lethargy, and stereotypy—domains that, in the present more methodologically conservative synthesis, did not reach significance. Conversely, the present analysis identified significant effects in Irritability and Inappropriate Speech, domains not highlighted as significant in earlier work. This divergence likely reflects differences in included studies, outcome harmonization strategy, and statistical correction (Hedges’ *g* with Knapp-Hartung adjustment) rather than a substantive disagreement in evidence and underscores the value of instrument-level harmonization in resolving domain-specific signal that single-instrument analyses may lack the power to detect.

The omega-3 dosages included in the present synthesis (200 mg–5.2 g/day) substantially exceed the upper bound reported across all seven prior meta-analyses in this field, which converge on a consistent range of approximately 200–1540 mg/day (mean doses 815–1320 mg/day) [2,3,8]. This upper extension is attributable to a single trial, Lundbergh (2022) [16], which administered 5.2 g/day—nearly 3.4 times the highest dose previously reported in this literature—and which also enrolled an adult population (mean age 28 years), the only such trial in the present synthesis. To assess whether this dosage and population outlier disproportionately influenced the pooled estimates, a sensitivity analysis excluding Lundbergh (2022) [16] was conducted for all affected domains (Supplementary Table S4). The pooled effect for Inappropriate Speech/Communication remained statistically significant following exclusion (t = −3.02, *p* = *0.014*), confirming that the beneficial effect on communication symptoms is robustly present within the pediatric population alone. The Irritability estimate was unchanged (t = −2.69, *p* = *0.043*), as Lundbergh did not contribute data to this domain. No changes in significance or direction were observed for Lethargy/Social Withdrawal (t = −1.01, *p* = 0.338), Hyperactivity (t = −0.19, *p* = 0.859), or Stereotypy (t = −1.49, *p* = 0.171). These results indicate that the dosage and population heterogeneity introduced by Lundbergh (2022) [16] did not drive the significant pooled effects reported in the primary analysis.

A critical methodological question concerns whether the domain-mapping strategy achieves genuine construct equivalence with the original ABC domains or whether it instead constructs broader behavioral categories that only partially correspond to what the ABC was designed to measure. We favor the latter, more conservative interpretation. The ABC domains were derived from factor-analytic work on a specific item set [15], whereas instruments such as SRS-2, GARS-2, and CBCL were developed under distinct theoretical frameworks, with subscales that overlap with—but are not isomorphic to—ABC constructs. For example, ABC Lethargy/Social Withdrawal denotes hypoactivity and reduced initiation, whereas SRS-2 Social Awareness assesses recognition of social cues, a related but conceptually narrower construct; pooling these under a single domain label therefore represents an assumption of behavioral-category convergence rather than a demonstration of measurement equivalence. This distinction matters clinically: a significant pooled effect on “Irritability” synthesized from ABC Irritability and SDQ Conduct Problems should be read as evidence for a broader aggression/dysregulation category responsive to omega-3 supplementation, not as confirmation that omega-3 specifically improves the item-level phenomena the original ABC Irritability subscale was designed to capture (e.g., self-injury, tantrums, and mood lability). We consider this reframing important for interpreting the present findings: the significant effects on Irritability and Inappropriate Speech/Communication should be understood as effects on multi-instrument behavioral constructs, and their translation to any single clinical instrument’s score should not be assumed without instrument-specific validation. This is consistent with, and further motivates, the limitations already discussed regarding domain-mapping validity (Section 4.2).

These results should nonetheless be interpreted within the constraints discussed in Section 4.2, particularly the modest k for Irritability and Hyperactivity, the reliance on clinical-judgment-based domain mapping across heterogeneous instruments, and the mixture of effect-size calculation routes. Larger, adequately powered primary trials using harmonized, ASD-specific outcome measures remain necessary to confirm whether the significant effects observed here for Irritability and Inappropriate Speech reflect genuine, clinically meaningful benefits of omega-3 supplementation.

### 4.1. Strengths

This meta-analysis presents several methodological strengths:

1. The review protocol was prospectively registered in PROSPERO prior to data collection, and study selection and data extraction were performed in duplicate with formal inter-rater reliability assessment.
2. Risk of bias was evaluated using the RoB 2 tool across all twelve included studies, providing domain-specific ratings aligned with current methodological standards [14].
3. The expansion beyond a single outcome instrument to eight validated scales (ABC, CGI-I, BASC-2, SRS/SRS-2, GARS-2, CARS/SDQ, VABS/PLS-4/PDDBI, CBCL), harmonized onto five common behavioral domains, substantially increased the evidence base (k = 6–12 per domain versus k = 4 in the ABC-only subset), improving precision without altering the direction of pooled effects, as confirmed by the sensitivity analysis (Section 3.6).
4. The use of Hedges’ *g* with small-sample bias correction and the conservative Knapp-Hartung adjustment, alongside reporting of 95% prediction intervals, represents appropriate and informative choices for meta-analyses with a limited number of studies per domain.
5. Publication bias was assessed using the PET-PEESE framework, a more robust approach to small-study effects than Egger’s test alone; combined with explicit documentation of the domain-mapping procedure (Supplementary Table S1) and the four distinct effect-size calculation routes employed, this supports both the credibility of the significant pooled effects observed for Irritability and Inappropriate Speech and the transparency and reproducibility of the extraction process.

### 4.2. Limitations

Several limitations must be acknowledged:

1. Domain-mapping validity. Mapping of non-ABC instruments onto ABC-derived domains relied on construct-content correspondence, following standard meta-analytic guidance for pooling standardized effect sizes across instruments measuring the same underlying construct [12], rather than on empirically validated cross-instrument equivalence (e.g., correlational or factor-analytic convergence between scales). This is a materially weaker form of justification: it assumes, rather than demonstrates, that instruments sharing a construct label are commensurable on the same standardized scale despite differing item content, response formats, and normative calibration. Consequently, pooling may combine measures that were not originally designed or validated to assess identical behavioral phenomena, potentially masking construct-level heterogeneity that Cochran’s Q and I^2^—which are sensitive primarily to statistical, not conceptual, heterogeneity—are not designed to detect. The negligible I^2^ observed in four of five domains should therefore not be interpreted as confirmation of measurement equivalence across instruments; it is equally consistent with genuine effect homogeneity and with a lack of statistical power, at the present k, to detect construct-level inconsistency.
2. Subscale selection. When an instrument offered multiple candidate subscales for the same domain (e.g., SRS/SRS-2 Social Awareness, Social Motivation, and Social Cognition for Lethargy/Social Withdrawal), a single subscale was selected a priori and applied consistently within that instrument to avoid averaging correlated subscales; this may not fully capture the construct as originally intended.
3. Heterogeneous effect-size derivation. Effect sizes were derived via four distinct routes (raw means/SD, CI-recovered SE, dichotomous responder data, and crossover Phase-1 data), varying in precision beyond what is reflected in reported I^2^. For Mankad (2015) [5], Satterthwaite-adjusted degrees of freedom (df = 31–51.2) were combined with nominal per-group n (18/19) in the absence of raw SDs, introducing study-specific imprecision.
4. Uneven domain coverage. No added instrument (including Voigt 2014 [30] and Mankad 2015 [5]) provided a subscale directly comparable to ABC Irritability, leaving this domain with the smallest expanded evidence base (k = 6); Hyperactivity was similarly underrepresented (k = 8).
5. Limited k per domain. The modest number of studies per domain, despite expansion, constrains the reliability of heterogeneity estimates, subgroup analyses, and publication-bias tests, particularly for Irritability and Hyperactivity.
6. Differential robustness across outcomes. Benefits on Inappropriate Speech remained robust across all sensitivity checks, whereas findings for Irritability and Stereotypy were sensitive to trial methodological quality, warranting cautious interpretation of these two outcomes specifically.
7. Hyperactivity instability. This domain showed non-negligible heterogeneity (I^2^ = 37.86%, τ^2^ = 0.080) and unstable PET-PEESE bias-corrected estimates, indicating lower robustness than the other four domains.
8. Crossover data restriction. Data from crossover trials (Parellada 2017 [15], Lundbergh 2022 [16]) were limited to Phase 1 rather than analyzed via Cochrane’s recommended paired approach [12] because neither trial reported the within-participant correlation coefficient necessary for a paired standardized mean difference, and imputing this parameter was judged too consequential to the effect-size magnitude to be justified. This reduced the effective sample size and statistical power for these two studies relative to what a full paired analysis would have afforded had the necessary correlation data been reported.
9. Clinical protocol heterogeneity. Omega-3/DHA-EPA dosage (200 mg–5.2 g/day), duration (4–52 weeks), and placebo composition varied substantially across trials, potentially introducing clinical heterogeneity not fully captured by subgroup moderators.
10. Publication-bias test power. Tests for publication bias have inherently limited power with fewer than ten studies; results for Irritability and Hyperactivity (k = 6, k = 8) should be interpreted with particular caution.
11. EPA:DHA composition. Formulations ranged from DHA-predominant (e.g., Voigt 2014 [30], Mazahery 2019/2020 [23]) to EPA-predominant (e.g., Amminger 2007 [20], Bent 2011 [21], Bent 2014 [22]). This heterogeneity precluded a dedicated EPA-versus-DHA subgroup meta-regression given limited k per domain; component-specific effects remain a priority for future primary research.
12. Safety data not synthesized. Adverse events were not systematically extracted from the included trials, as data extraction focused on behavioral efficacy outcomes. The comparison drawn between omega-3’s safety profile and that of pharmacological agents (Section 4) is therefore based on external literature rather than on a systematic within-review safety synthesis; a dedicated safety analysis of the included trials would be needed to substantiate this comparison empirically.

## 5. Conclusions

The clinical magnitude of the significant effects remains modest and considerably smaller than that reported for FDA-approved pharmacological agents, but given the favorable safety profile of omega-3 supplementation, these results provide a more precise, hypothesis-supporting basis than previously available for considering omega-3 as a low-risk adjunctive option for the associated behavioral domain of inappropriate speech/communication assessed here—not as evidence of improvement in core ASD symptomatology. Domain mapping across instruments relied on clinical judgment rather than validated psychometric equivalence, and coverage remained uneven across domains, a limitation that adequately powered, ASD-specific primary trials should address. Future research should prioritize standardized outcome reporting, larger samples targeting Irritability and Hyperactivity specifically, and pre-registered protocols to confirm whether the effects identified here reflect genuine, clinically meaningful benefit on these associated behavioral domains, distinct from the core social-communication and restricted/repetitive-behavior deficits that define ASD diagnostically.

## Figures and Tables

**Table 1:** Studies characteristics. Gender is specified as a percentage of males, and duration of intervention as time (weeks).

			Intervention		Placebo					
Author, Year	Country	*N*	Years Mean (SD)	%	Years Mean (SD)	%	EPA:DHA	Dosage (Daily)	Time	Placebo
Amminger, 2007 [20]	AUT	13	10.5 (3.2)	100	12.1 (2.7)	100	1.2:1	1.54 g (840 mg EPA + 700 mg DHA)	6	Coconut oil
Johnson, 2010 [29]	USA	23	3.73 (0.64)	-	3.23 (0.71)	-	-	400 mg DHA	12	Health diet
Bent, 2011 [21]	USA	27	5.85 (1.83)	-	5.817 (1.417)	-	1.5:1	1.1 g (700 mg EPA + 460 mg DHA)	12	Safflower oil
Bent, 2014 [22]	USA	57	7.35 (1.025)	89.7	7.083 (1.1)	85.7	1.5:1	1.1 g (700 mg EPA + 460 mg DHA)	6	Safflower oil
Voigt, 2014 [30]	USA	48	5.8 (1.8)	83	6.5 (2.2)	83	-	200 mg DHA	24	Corn + soybean oil
Mankad, 2015 [5]	CAN	38	3.5 (-)	77.7	3.9 (-)	68.4	3:1	1.5 g (1125 mg EPA + 375 mg DHA)	24	Olive oil
Parellada, 2017 [15]	ESP	68	9.72 (3.64)	84	9.72 (3.64)	84	1.5:1	577.5 mg EPA + 385 mg DHA (5–11 years old); 693 mg EPA + 462 mg DHA (12–17 years old)	8	Liquid paraffin + vitE
Mazahery, 2019 [23]	NZL	39	4.8 (1.5)	78	5.7 (1)	81	-	722 mg DHA	52	Olive oil + alpha tocopherol
Mazahery, 2020 [23]	NZL	31	5.3 (1.4)	84	5.3 (1.4)	84	-	722 mg DHA	52	Olive oil + alpha tocopherol
Doaei, 2021 [31]	IRN	54	8.1 (6.7)	67.9	8.2 (3.6)	76.9	1.5:1	1 g (180 mg EPA + 120 mg DHA)	8	Medium chain triglyceride
De la Torre-Aguilar, 2022 [28]	ESP	54	3.80 (0.93)	74	3.49 (0.93)	88	1:32	0.825 g (25 mg EPA + 800 mg DHA)	24	Sunflower oil
Lundbergh, 2022 [16]	DNK	21	28 (7)	50	28 (7)	50	1.5:1	5.2 g (2.4 g EPA + 1.6 g DHA)	4	Sunflower oil

**Table 2:** Subgroup metanalysis by outcome.

Outcome	Intervention Dose		Age		Intervention Duration	
	*Q_M*	*p*	*Q_M*	*p*	*Q_M*	*p*
Irritability	4.84	*0.* *028*	4.27	*0.* *039*	4.27	*0.039*
Lethargy/Social Withdrawal	1.74	0.187	0.44	0.508	1.34	0.248
Hyperactivity	3.03	0.082	1.99	0.158	1.99	0.158
Stereotypy	0.18	0.674	1.02	0.312	0.42	0.517
Inappropriate Speech	0.31	0.576	1.54	0.214	4.39	*0.036*

**Table 3:** Summary of Meta-Analytic Results for the Five ABC Subscales according to RE = Random-effects (REML, Knapp-Hartung); FE = Fixed-effects (inverse variance). Δ*g* = absolute difference between models. *g* = Hedges’ *g* (pooled effect size); CI = confidence interval; PI = prediction interval. Negative values of *g* indicate a benefit for the intervention group.

Outcome	RE *g*	RE 95% CI	95% PI	FE *g*	FE 95% CI	Δ*g*
Irritability	−0.287	[−0.562, −0.013]	[−0.562, −0.013]	−0.29	[−0.56, −0.01]	<0.01
Social Withdrawal/Lethargy	−0.082	[−0.261, 0.098]	[−0.261, 0.098]	−0.08	[−0.26, 0.10]	<0.01
Hyperactivity	−0.066	[−0.485, 0.354]	[−0.853, 0.722]	−0.05	[−0.44, 0.34]	0.016–0.02
Stereotypy	−0.118	[−0.278, 0.041]	[−0.278, 0.041]	−0.12	[−0.28, 0.04]	<0.01
Inappropriate Speech	−0.213	[−0.363, −0.063]	[−0.363, −0.063]	−0.21	[−0.363, −0.063]	<0.01

## Data Availability

The original contributions presented in this study are included in the article/Supplementary Material. Further inquiries can be directed to the corresponding author.

## Supplementary Materials

The following supporting information can be downloaded at: https://www.mdpi.com/article/10.3390/nu18172821/s1, Figure S1: Traffic light plot from RoB-2; Figure S2: Irritability Forest Plot for ABC subscales; Figure S3: Lethargy/Social Withdrawal Forest Plot for ABC Subscales; Figure S4: Hyperactivity Forest Plot for ABC subscales; Figure S5: Stereotypy Forest Plot for ABS subscales; Figure S6: Inappropriate Speech Forest Plot for ABC subscales; Table S1: Instrument-to-ABC-Domain Mapping for Non-ABC Studies; Table S2: Summary of Meta-Analytic Results for the Five ABC Subscales according to RE = Random-effects (REML, Knapp-Hartung); FE = Fixed-effects (inverse variance); Table S3: PET-PEESE test of publication bias by domain; Table S4: Exclusion Sensitive analysis. References [5,15,16,20,21,22,23,28,29,30,31] are cited in the supplementary materials.

## Abbreviations

The following abbreviations are used in this manuscript:

ASD	Autism Spectrum Disorder
DSM	Diagnostic and Statistical Manual of Mental Disorders
RRIB	Restricted, Repetitive Patterns of Behavior, Interests, or Activities
PUFAs	polyunsaturated fatty acids
DHA	docosahexaenoic acid
EPA	eicosapentaenoic acid
ABC	Aberrant Behavior Checklist (or Autism Behavior Checklist)
CARS	Childhood Autism Rating Scale
GARS-2	Gilliam Autism Rating Scale—Second Edition
SRS/SRS-2	Social Responsiveness Scale/Social Responsiveness Scale—Second Edition
PDDBI	Pervasive Developmental Disorder Behavior Inventory
CGI-I	Clinical Global Impressions—Improvement scale
BASC-2	Behavioral and Emotional Screening System (or Behavior Assessment System for Children—Second Edition)
CBCL	Child Behavior Checklist
SDQ	Strengths and Difficulties Questionnaire
VABS	Vineland Adaptive Behavior Scales
PLS-4	Preschool Language Scale—Fourth Edition

## Appendix A

### Appendix A.1. The Complete Search Strategy for PubMed Is Listed Below

((((((“Fatty Acid, omega-3”[mh]) OR “eicosa$ Acid”[mh]) OR “Docosahexaenoic Acids”[mh]) OR “Linolenic Acid”[mh]) OR (((((((“omega 3”[tiab]) OR (“omega-3 fatty acid”[tiab])) OR (“Eicosapentaenoic Acid”[tiab])) OR (“Docosahexaenoic Acids”[tiab])) OR (“Linolenic Acid”[tiab])) OR (“lipoic acid”[tiab])) OR (“ethyl-eicosapentaenoic acid”[tiab]))) AND (((“Autism Spectrum Disorder”[mh]) OR ((((((“autism”[tiab]) OR (“autism spectrum disorder”[tiab])) OR (“ASD”[tiab])) OR (“Asperger”[tiab])) OR (“Pervasive development disorder”[tiab])) OR (“PPD”[tiab])))))).

### Appendix A.2. Complete Search Strategy for Other Scientific Reports Data Bases

All search strategies used in the remaining databases were based on the one presented in Appendix A.1, according to the rules of each database. However, the main terms are the same as those used for Pubmed.

### Appendix A.3. Search Strategy for Not Published Trials Ongoing

None of the authors of the clinical trials searched on ClinicalTrials.gov responded to the request for information regarding ongoing studies or those with results.
