# Supplementary material for: Does Omega-3 Help After All in Autism Spectrum Disorder? An Update Systematic Review and Meta-Analysis of Randomized Trials
Source: Nutrients. 2026 Aug 28;18(17):2821. doi: 10.3390/nu18172821 (PMC13567361; doi:10.3390/nu18172821)
Supplement: Supplementary file 1 [file nutrients-18-02821-s001.zip › nutrients-4473531-supplementary.pdf]

Supplementary Figure S1. Traffic light plot from RoB-2

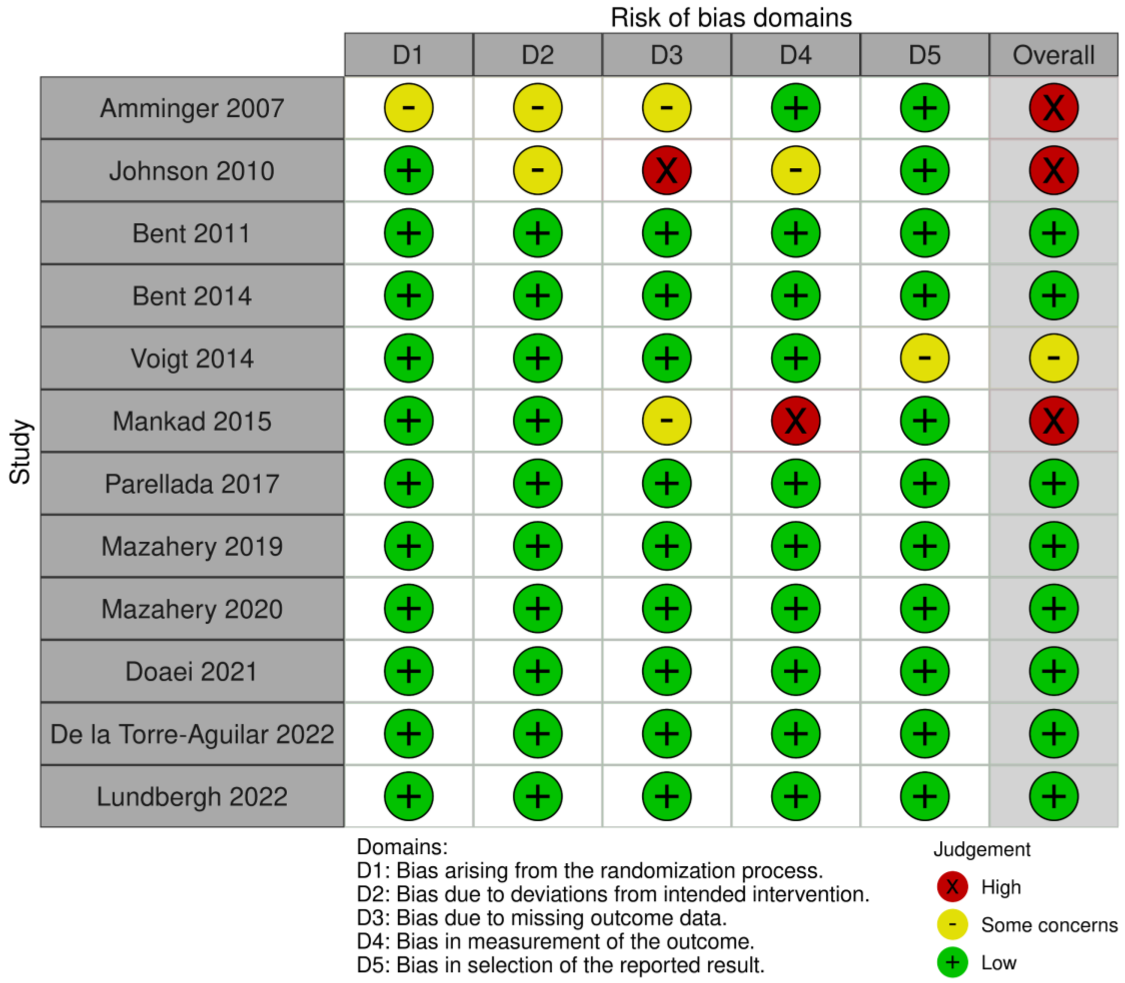

Supplementary Table S1. Instrument-to-ABC-Domain Mapping for Non-ABC Studies

Author, Year	Instrument	Original subscale/item	ABC domain assigned	Mapping rationale
Johnson, 2010	CBCL	Withdrawn	Lethargy/Social Withdrawal	Direct construct equivalence
Johnson, 2010	CBCL	Aggression	Irritability	Captures aggressive/tantrum behavior, core to Irritability
Johnson, 2010	CBCL	Attention Problems	Hyperactivity	Closest available proxy; CBCL lacks a pure hyperactivity subscale
Johnson, 2010	CBCL	PDD	*Excluded (global composite)*	Composite index, not domain-specific
Voigt, 2014	CGI-I	Social interaction	Lethargy/Social Withdrawal	Social interaction/withdrawal construct overlap
Voigt, 2014	CGI-I	Communication	Inappropriate Speech	Only CGI-I domain addressing language/speech

Voigt, 2014	CGI-I	Stereotyped behavior	Stereotypy	Direct construct equivalence
Voigt, 2014	CGI-I	Overall behavior	*Excluded (global composite)*	Composite/global impression, not domain-specific
Mankad, 2015	BASC-2	Externalizing Problems	Hyperactivity	Aggregates impulsivity/hyperactivity items
Mankad, 2015	BASC-2	Internalizing Problems	*Not used (see PDDBI row below)*	Superseded by PDDBI Ritualism for Stereotypy in final selection
Mankad, 2015	PDDBI	Autism composite	*Excluded (global composite)*	Composite index
Mankad, 2015	VABS	Adaptive Functioning Composite	*Excluded (global composite)*	Global adaptive index; inverted-direction scale
Mankad, 2015	PLS-4	Total language score	Inappropriate Speech	Language/communication construct
Parellada, 2017	SRS	Social Awareness	Lethargy/Social Withdrawal	Selected as representative social-withdrawal proxy over Social Cognition/Motivation
Parellada, 2017	SRS	Social Communication	Inappropriate Speech	Communication construct
Parellada, 2017	SRS	Autistic Mannerisms	Stereotypy	Direct construct equivalence
Parellada, 2017	SRS Total; CGI severity	—	*Excluded (global composite)*	Global/composite measures
Mazahery, 2020	SRS	Social awareness	Lethargy/Social Withdrawal	Same criterion as Parellada, 2017 (consistency across SRS studies)
Mazahery, 2020	SRS	Communication	Inappropriate Speech	Communication construct
Mazahery, 2020	SRS	Repetitive/restricted interest & behaviour	Stereotypy	Direct construct equivalence
Mazahery, 2020	SRS Total; Social communicative functioning; Social cognition; Social motivation	—	*Excluded (redundant/global)*	Overlap with assigned subscales or composite nature
Doaei, 2021	GARS-2	Stereotyped Behaviors	Stereotypy	Direct construct equivalence
Doaei, 2021	GARS-2	Social Communication	Inappropriate Speech	Communication construct
Doaei, 2021	GARS-2	Social Interaction	Lethargy/Social Withdrawal	Social interaction/withdrawal construct overlap
Doaei, 2021	GARS-2 Total	—	*Excluded (global composite)*	Composite index
De la Torre-Aguilar, 2022	SDQ	Hyperactivity	Hyperactivity	Direct construct equivalence
De la Torre-Aguilar, 2022	SDQ	Conduct problems	Irritability	Aggression/conduct construct overlap
De la Torre-Aguilar, 2022	PDDBI	Ritual score	Stereotypy	Direct construct equivalence
De la Torre-Aguilar, 2022	PDDBI	Semantic pragmatic problems	Inappropriate Speech	Language/pragmatics construct
De la Torre-Aguilar, 2022	PDDBI	Social approach behaviors	Lethargy/Social Withdrawal	Social approach/withdrawal construct overlap

De la Torre-Aguilar, 2022	CARS	—	*Excluded (global composite)*	Global severity measure
De la Torre-Aguilar, 2022	Battelle subscales	—	*Excluded*	Inverted-direction adaptive-functioning scale; not used in domain pooling
Lundbergh, 2022	Conners	Hyperactivity and impulsivity	Hyperactivity	Direct construct equivalence
Lundbergh, 2022	SRS-2	RI&RB	Stereotypy	Direct construct equivalence
Lundbergh, 2022	SRS-2	Communication and interaction	Inappropriate Speech	Communication construct
Lundbergh, 2022	SRS-2	Social awareness	Lethargy/Social Withdrawal	Same criterion as Parellada/Mazahery 2020
Lundbergh, 2022	SRS-2 Total; BRIEF-A subscales	—	*Excluded (global/executive function, not ASD-specific)*	Outside ABC construct scope

Supplementary Table S2. Summary of Meta-Analytic Results for the Five ABC Subscales according to RE = Random-effects (REML, Knapp-Hartung); FE = Fixed-effects (inverse variance). Δg = absolute difference between models. *g* = Hedges' *g* (pooled effect size); CI = confidence interval; PI = prediction interval. Negative values of *g* indicate a benefit for the intervention group.

Outcome	RE *g*	RE 95% CI	95% PI	FE *g*	FE 95% CI	Δg
Irritability	−0.249	[−0.765, 0.267]	[−0.776, 0.278]	−0.249	[−0.765, 0.267]	< 0.01
Lethargy/Social Withdrawal	−0.129	[−0.494, 0.235]	[−0.494, 0.235]	−0.129	[−0.494, 0.235]	< 0.01
Hyperactivity	−0.183	[−0.841, 0.476]	[−0.841, 0.476]	−0.183	[−0.841, 0.476]	< 0.01
Stereotypy	−0.077	[−0.394, 0.241]	[−0.394, 0.241]	−0.077	[−0.394, 0.241]	< 0.01
Inappropriate Speech	−0.204	[−0.538, 0.130]	[−0.538, 0.130]	−0.204	[−0.538, 0.130]	< 0.01

Supplementary Figure S2. Irritability Forest Plot for ABC subscales.

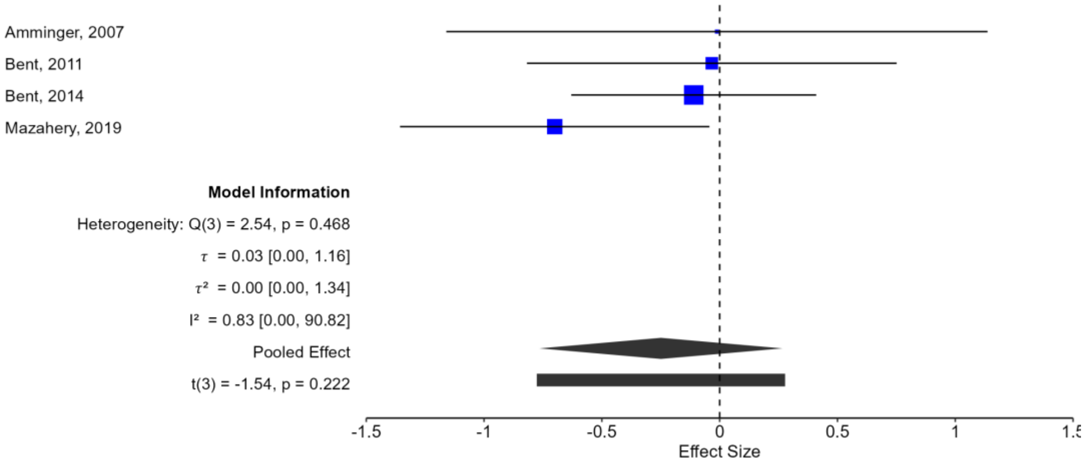

Supplementary Figure S3. Lethargy/Social Withdrawal Forest Plot for ABC Subscales.

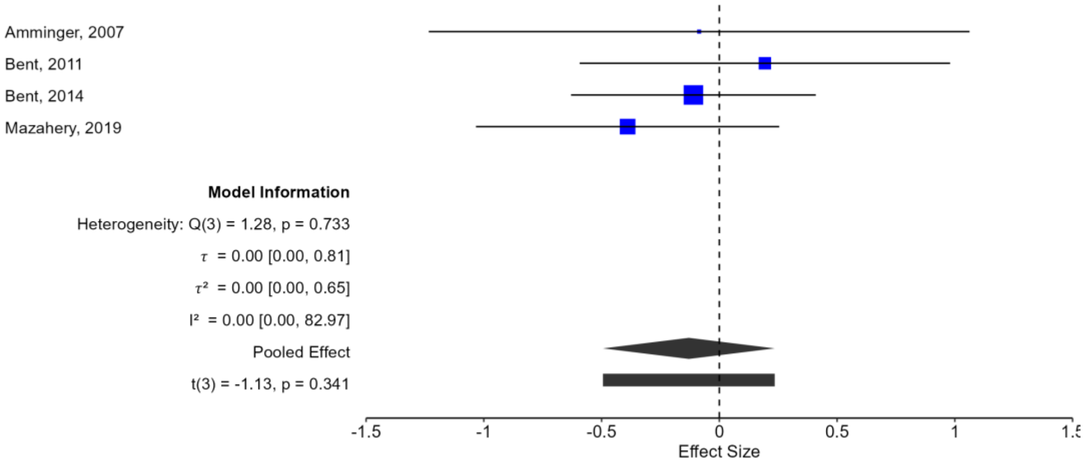

Supplementary Figure S4. Hyperactivity Forest Plot for ABC subscales

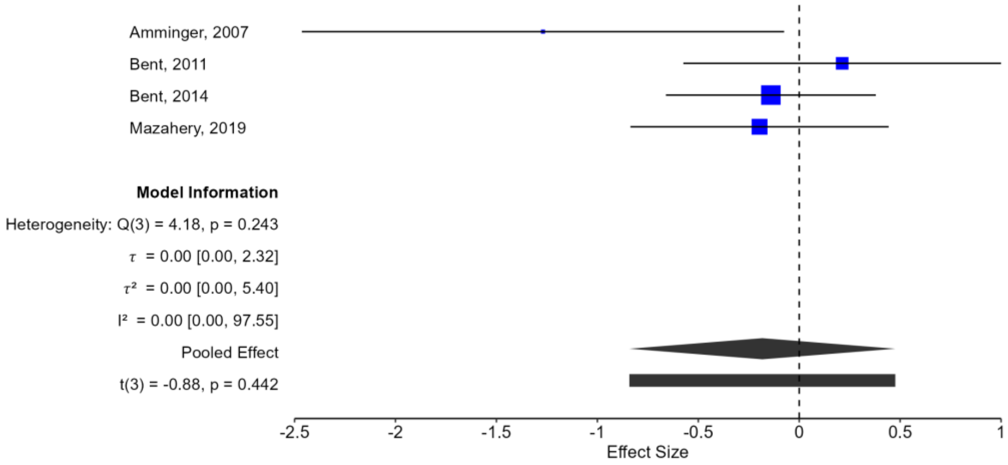

Supplementary Figure S5. Stereotypy Forest Plot for ABS subscales.

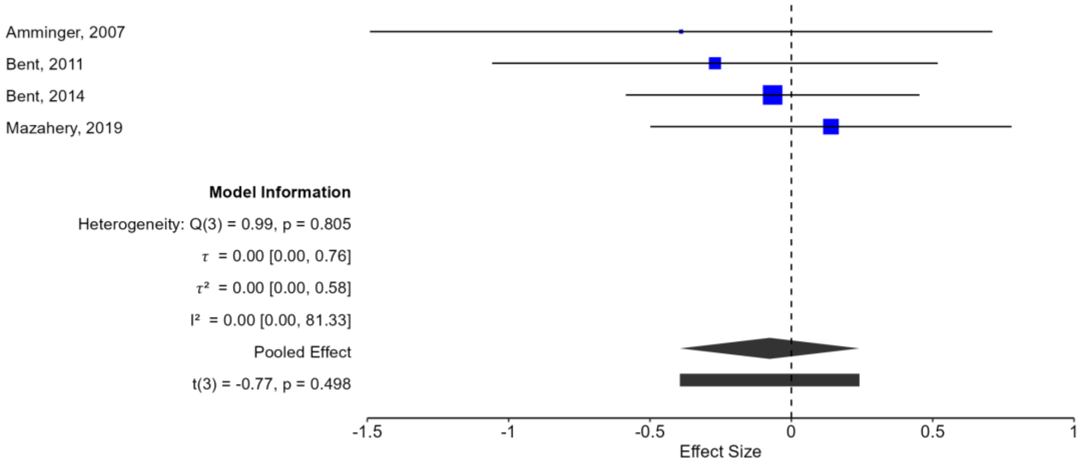

Supplementary Figure S6. Inappropriate Speech Forest Plot for ABC subscales.

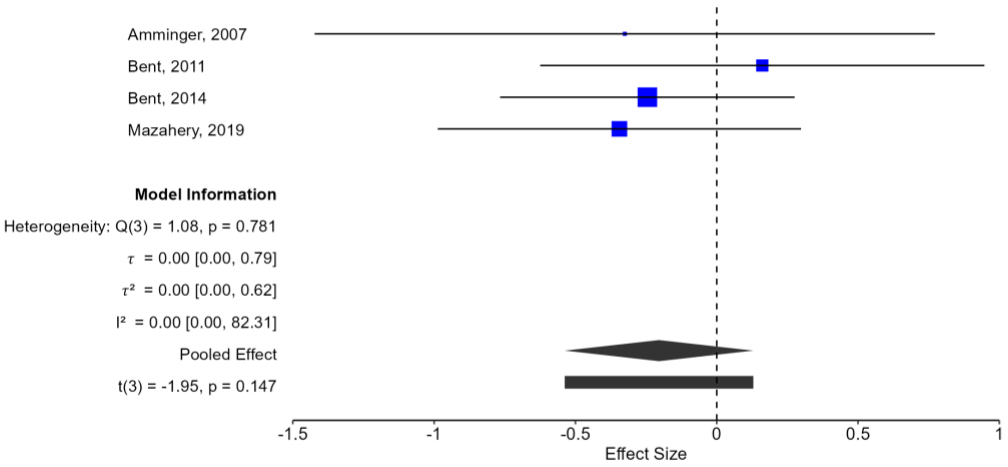

Supplementary Table S3. PET-PEESE test of publication bias by domain.

Outcome	k	Test of Publication Bias (PET slope) t	df	p	PET-corrected *g*	PEESE-corrected *g*
Irritability	6	0.419	4	.696	−0.492	−0.403
Lethargy/Social Withdrawal	12	1.329	10	.213	−0.515	−0.260
Hyperactivity	8	−1.272	4	.272	+0.501	+0.178
Stereotypy	11	−1.159	9	.276	+0.191	+0.030
Inappropriate Speech	11	1.527	9	.161	−0.633	−0.379

Supplementary Table S4. Exclusion Sensitive analysis

Excluded Studies	*g*	*p*
Lundbergh, 2022		
Irritability	-2.69	*.043**
Social Withdraw	-1.01	.338
Hyperactivity	-0.19	.859
Stereotypy	-1.49	.171
Inappropriate Speech	-3.02	*.014**
High risk at RoB-2 (Mankad,2015; Johnson, 2010; Amminger, 2007)	-2.17	.118
Irritability	-2.17	.062
Social Withdraw	-1.79	.147
Hyperactivity	-3.01	*.017**
Stereotypy	-2.64	*.030**
Inappropriate Speech		
